# Clinical Characteristics of Secondary Immune Thrombocytopenia Associated With Primary Sjögren's Syndrome

**DOI:** 10.3389/fmed.2020.00138

**Published:** 2020-04-17

**Authors:** Fan Dai, Guomei Yang, Peishi Rao, Puqi Wu, Rongjuan Chen, Yuechi Sun, Yun Peng, Hongyan Qian, Bin Wang, Shiju Chen, Yuan Liu, Guixiu Shi

**Affiliations:** ^1^Department of Rheumatology and Clinical Immunology, The First Affiliated Hospital of Xiamen University, Xiamen, China; ^2^School of Medicine, Xiamen University, Xiamen, China; ^3^Xiamen Key Laboratory of Rheumatology and Clinical Immunology, Xiamen, China

**Keywords:** primary Sjögren's syndrome, immune thrombocytopenia, platelet, interstitial lung diseases, arthritis

## Abstract

**Objective:** Clinical characteristics of immune thrombocytopenia (ITP) associated with primary Sjögren's syndrome (pSS) have not been clearly defined. This study aimed to evaluate the prevalence and clinical characteristics of secondary ITP in patients with pSS.

**Methods:** 291 pSS patients in our hospital were retrospectively analyzed. Clinical manifestations and laboratory findings were compared between pSS patients with and without ITP.

**Results:** The prevalence of secondary ITP in pSS patients was 12.03%. Compared to pSS patients without ITP, pSS patients with ITP were younger and had higher disease activity. The prevalence of interstitial lung diseases (ILD) was significantly lower in pSS patients with ITP (30.43 vs. 54.95%; *P* = 0.029), and it was the same with arthritis (17.14 vs. 3.9.11%; *P* = 0.014) and dry eye (33.33 vs. 54.17%, *P* = 0.027) compared with those without ITP. Serum creatinine level was lower in pSS patients with ITP (*P* = 0.009), while positivity of anti-histone autoantibodies was higher in pSS patients with ITP (*P* = 0.025).

**Conclusion:** This study is an initial report describing clinical features of ITP in pSS. The lower incidence of ILD and arthritis among pSS patients with ITP indicated potential active roles of platelets in the pathogenesis of fibrosis or inflammatory arthritis, which may open the way for further experimental and clinical work.

## Introduction

Primary Sjögren's syndrome (pSS) is a common systemic autoimmune disease characterized by lymphocytes infiltrating in the exocrine glands including salivary and lacrimal glands. The hallmark clinical symptoms of pSS are dryness of the mouth and eyes (1). Some patients may present diverse extra glandular manifestations such as inflammatory arthritis and interstitial lung diseases (2).

Immune thrombocytopenia (ITP) is an immune-mediated disorder characterized by increased platelet destruction or impaired platelet production, resulting in platelet count >100,000 per cubic millimeter and varying degree of bleeding risk (3). ITP is commonly associated with autoimmune diseases, especially with systemic lupus erythematosus (SLE). The prevalence, clinical characteristics and prognostic impact of ITP in SLE have been well-studied (4, 5), providing many insights in clinical management of secondary ITP associated with SLE. Presence of ITP in pSS has been reported by some case reports (6, 7). However, those case reports are insufficient to fully explore the clinical features of secondary ITP in pSS. Currently, our knowledge about secondary ITP in pSS are still limited. Hence, to investigate the prevalence, clinical characteristics, and immunological features of secondary ITP in patients with pSS, we performed this retrospective study.

## Patients and Methods

### Study Subjects

This study was approved by Clinical Research Ethics Committee of the First Affiliated Hospital of Xiamen University (KY2016-001) and patient's informed consent to publish was obtained. All available medical records of patients between January 2015 and January 2018 in inpatient department of rheumatology were retrospectively reviewed. A total of 291 patients with diagnosis of pSS were included. The diagnosis of pSS was based on the 2002 American-European Consensus Group criteria for pSS (8). All secondary ITP patients fulfilled the diagnosis criteria according to the American Society of Hematology guidelines (3).

### Data Collection

Clinical data including detailed patient history, laboratory findings and treatment strategy were obtained from patients' medical records of the first encounter. Presence of clinical manifestations related to pSS were analyzed. Symptoms related to bleeding such as skin and mucous membrane petechiae were analyzed in pSS patients with ITP. Laboratory findings and immunological characteristics including erythrocyte sedimentation rate (ESR), C-reactive protein (CRP), complement 3 (C3), complement 4 (C4), immunoglobulins, autoantibodies were also analyzed. In addition, disease activity of pSS in all patients were determined by using the European League Against Rheumatism Sjögren's Syndrome Disease Activity Index (ESSDAI) (9). The severity of hemorrhagic manifestations of the patients were described by ITP specific bleeding assessment tool (ITP-BAT) (10).

### Statistical Analysis

Continuous data were expressed as mean with standard deviation (SD) for normally distributed data, and median with 25–75th percentiles (Q25–Q75) for non-normally distributed data. The single-sample K-S (Kolmogorov-Smirnov) test was used to detect whether the data obey normal distribution. *T-*test was used for continuous data with normal distribution, while Mann Whitney *U*-test was used for continuous data that did not obey normal distribution. Categorical data were expressed as positive number/test number (%). The Chi-squared test or Fisher exact test was used to compare the binary data. *P* < 0.05 were considered statistically significant. Logistic regression analysis was further performed to identify risk factors associated with ITP in pSS patients. Odds ratio (OR) with 95% confidence interval (95%CI) was calculated in the logistic regression analysis. All analyses were performed using SPSS software.

## Results

### Basic Characteristics of pSS Patients With ITP

A total of 291 patients with pSS were analyzed. Among those patients, 35 pSS patients were complicated with secondary ITP, with a prevalence of 12.03%. 16 (45.71%) pSS-ITP patients had very low level of platelets (<20 × 10^9^/L) at the time of first diagnosis. Bleeding symptoms were presented in 23 (65.71%) patients with pSS-ITP and no one had symptoms of intracranial hemorrhage. Eleven of them were rated S2, and the main hemorrhagic manifestations were petechiae and ecchymoses of the extremities. 12 patients were rated M1, 10 of whom had symptoms of gum bleeding. Only 1 patient developed symptoms of organ bleeding, showing symptoms of hematuria. Glucocorticoids (GCs) were used in all ITP patients, and 12 (34.29%) patients were resistant to GCs therapy, which was defined as platelet count remaining <30 × 10^9^/L or <2-fold increase compared to baseline platelet count following 4 weeks of GCs treatment (3, 11).

### Clinical Characteristics of pSS Patients With ITP

Clinical characteristics of pSS patients with ITP are shown in Table 1. Compared with pSS patients without ITP, those pSS patients with ITP were younger at the time of pSS diagnosis (*P* = 0.006). Besides, the disease activity of pSS was higher in pSS patients with ITP (*P* = 0.013). The prevalence of ILD was lower in pSS patients with ITP (30.43 vs. 54.95%; *P* = 0.029), and it was the same with arthritis (17.14 vs. 39.11%; *P* = 0.014). The presence of dry eye was also less common in pSS patients with ITP compared those without ITP (33.33 vs. 54.17%, *P* = 0.027). There was no obvious difference in the presence of other clinical manifestations including weight loss and fever. The laboratory findings of patients with and without ITP were also compared. Concentration of serum creatinine was lower in pSS patients with ITP (*P* = 0.009), as shown in Table 1. No significant differences were detected between pSS patients with and without ITP in other parameters such as leukocyte count in periphery blood, hemoglobin level or parameters associated with liver function.

**Table 1:** Comparison of clinical characteristics between pSS patients with and without ITP.

	**pSS with ITP** ** (*n* = 35)**	**pSS without ITP** ** (*n* = 256)**	***P*-value**
**Basic characteristics**			
Age at diagnosis (years)	40.09 ± 15.74	46.83 ± 13.29	0.006
Gender (female/male)	32/3	234/22	0.736
**Clinical manifestations**			
Dry mouth (%)	19/33 (57.58)	169/240 (70.42)	0.161
Dry eye (%)	11/33 (33.33)	130/240 (54.17)	0.027
Arthritis (%)	6/35 (17.14)	97/248 (39.11)	0.014
Interstitial lung disease (%)	7/23 (30.43)	122/222 (54.95)	0.029
ESSDAI	5.00 (3.00–7.00)	4.00 (2.00–6.00)	0.013
**Laboratory findings**			
Platelet, × 10^9^/L	35.00 (10.00–81.00)	232.00 (196.00–288.5)	<0.001
Creatinine, mg/dL	46.00 (37.00–58.00)	54.00 (45.00–53.25)	0.009
ESR, mm/h	23.00 (12.50–58.50)	30.00 (14.00–53.00)	0.888
C-reactive protein, mg/L	1.66 (0.28–4.33)	2.01 (0.54–7.95)	0.184
Leukocyte, × 10^9^/L	6.30 (4.16–9.56)	5.69 (4.47–7.79)	0.260
Hemoglobin, g/L	120.00 (107.00–136.00)	125.00 (111.00–134.00)	0.517
ALT, U/L	20.50 (15.00–27.75)	18.00 (13.00–28.00)	0.221
AST, U/L	23.50 (33.25–15.00)	19.00 (15.00–27.00)	0.185

*Data are presented as mean ± SD (standard deviation) or median with 25–75th percentiles, positive number/tested number (%). ESSDAI, European League Against Rheumatism Sjögren's Syndrome Disease Activity Index; ESR, erythrocyte sedimentation rate; ALT, alanine aminotransferase; AST, aspartate aminotransferase*.

### Immunological Features of pSS Patients With ITP

A comparison of immunological features between pSS patients with and without ITP is shown in Table 2. Though it seemed that level of C4 and IgA level were numerically lower in pSS patients with ITP when compared with pSS patients without ITP, the difference was not statistically significant. No significant differences were found between those two groups in percentages of T cells as well as the subsets. Among autoantibodies, positivity of anti-histone antibodies (AHA) was significantly higher in pSS patients with ITP (*P* = 0.025), while no significant differences were detected in other autoantibodies such as anti-SSA, anti-SSB (data not shown in Table 2).

**Table 2:** Comparison of immunological characteristics between pSS patients with and without ITP.

	**pSS with ITP** ** (*n* = 35)**	**pSS without ITP** ** (*n* = 256)**	***P*-value**
Complement 3, mg/dL	0.94 (0.81–1.13)	0.98 (0.86–1.13)	0.445
Complement 4, mg/dL	0.15 (0.13–0.24)	0.20 (0.15–0.25)	0.054
Immunoglobulin A, g/L	2.56 (1.75–3.51)	2.92 (2.21–2.78)	0.083
Immunoglobulin M, g/L	17.00 (13.45–20.50)	15.70 (13.00–20.58)	0.700
Immunoglobulin G, g/L	1.36 (0.85–2.36)	1.17 (0.84–1.60)	0.193
CD3^+^ T cells (%)	71.41 ± 5.78	72.43 ± 12.00	0.871
CD3^+^CD4^+^ T cells (%)	36.41 ± 13.56	42.01 ± 10.08	0.359
CD3^+^CD8^+^ T cells (%)	27.55 ± 10.00	27.84 ± 9.62	0.957
Positive ANA (%)	25/32 (78.12)	189/232 (81.47)	0.634
Positive AHA (%)	3/31 (9.68)	3/224 (1.34)	0.025

*Data are presented as median with 25–75th percentiles, positive number/tested number (%). ANA, antinuclear antibodies; AHA, anti-histone antibodies*.

### Potential Risk Factors of ITP Development in pSS Patients

To identify the risk factors that might be used to predict the development of ITP in pSS patients, logistic regression analysis was then performed. Variables including age, gender and those significantly different between pSS patients with and without ITP were included in regression analysis. The results suggested that the presence of arthritis was negatively associated with ITP in pSS patients (OR = 0.281, 95%CI 0.082–0.969; *P* = 0.044) (Table 3). Other factors such as sex and age of pSS diagnosis were not significantly related to the development of ITP in pSS patients (Table 3).

**Table 3:** Analysis of risk factors associated with ITP among pSS patients.

**Variables**	**OR**	**95%CI**	***P*-value**
Sex (Female)	2.982	0.458–19.415	0.253
Age of pSS diagnosis	1.020	0.981–1.061	0.320
Interstitial lung disease	0.432	0.141–1.329	0.143
Arthritis	0.281	0.082–0.969	0.044
Positive AHA	8.131	0.78–84.722	0.080
Dry eye	0.629	0.212–1.860	0.402
Creatinine	1.022	0.992–1.053	0.152
Immunoglobulin G	0.777	0.513–1.178	0.235
Complement 3	0.996	0.906–1.096	0.935

*AHA, anti-histone antibodies; OR, odds ratio; 95%CI, 95% confidence interval*.

## Discussion

ITP has been overlooked in patients with pSS for many years, and our knowledge about it is still limited. This study was the first to explore the overall prevalence, specific clinical and immunological characteristics of ITP in patients with pSS. Our results showed that the prevalence of ITP in patients with pSS was 12.03%, suggesting that secondary ITP was common in pSS patients. pSS patients with ITP were younger at the diagnosis of pSS and had higher disease activity, suggesting young pSS patients with high disease activity might be more prone to develop ITP. In addition, the development of ITP might be a protective factor for arthritis and ILD development, which indicated potential active roles of platelets in the pathogenesis of inflammatory arthritis or fibrosis.

Autoimmune disease (AID) is often complicated with ITP, while detailed mechanisms of platelet destruction are still unclear (12). Pathogenetic mechanisms of primary ITP comprise both, autoantibody- (such as autoantibodies to platelet and thrombopoietin) and T cell-mediated immune responses (13). A study reported that platelet destruction was part of an immune complex-mediated destruction of the reticulo-endothelial system in SLE (14). It has been suggested that elevated plasma P-selectin autoantibodies might play a role in the pathogenesis of ITP in pSS patients (15). Chen et al. pointed out that the expression of FcγRIIb on B cells was significantly decreased in patients with pSS with severe thrombocytopenia. After high-dose of hormone treatment, the expression of FcγRIIb on memory B cells was up-regulated, and the platelet count was significantly increased (16). Those studies are suggestive of a role of the humoral immune response in platelet destruction which may occur in pSS. However, which autoantibodies presumably contribute to platelet destruction and how T cells participate in ITP in pSS remains to be investigated.

Platelets are small, circulating, anucleate cells that are derived from megakaryocytes. In addition to their common function in the regulation of thrombosis, it has been recognized that platelets also have multiple functions in regulating innate and adaptive immunity (17). Evidence about the effects of platelets in promoting inflammation in inflammatory arthritis have been demonstrated by several studies in animal models (18). Besides platelets itself, platelets derived microparticles were also important in the pathogenesis of inflammatory arthritis (19). Those studies suggest that platelet might be an active player in promoting inflammatory arthritis. Our data showed that the prevalence of arthritis was significantly lower in pSS patients with ITP whose platelets decreased significantly, which provided new evidence for the potential active role of platelets in the pathogenesis of inflammatory arthritis.

Fibrosis of organs such as lung is a common complication associated with autoimmune diseases, and effective treatment strategies are still limited. Though researchers have devoted lots of effort in exploring the mechanisms involved in fibrosis and uncovering possible treatment targets, our understanding is still limited. It is hard to fully reveal the underlying mechanisms of fibrosis in autoimmune diseases just on the aspects of immune cells because multiple factors are synergistically involved in it. In recent years, the role of platelets in fibrosis have also been found by some studies. Platelets were capable of storing a number of biologically active molecules in intracellular granules, several of which have been proven to promote the activation of fibroblast such as serotonin and platelet-derived growth factor (20, 21). Our data showed that, in pSS patients with ITP, the prevalence of ILD was significantly lower, suggesting a possible role of platelets during the development of ILD.

There were several obvious limitations in this study. First, this was a retrospective study, some clinical manifestations may change over time. Second, the sample size was small, and patients were enrolled from a single center, the lack of certain clinical information of some patients may lead to the bias of this study. Third, experimental data are lacking to support assumptions based on our results. The results in our study needed to be validated by multi-center prospective studies in the future.

In summary, this is an initial report describing clinical features of ITP in pSS, which may open the way for further experimental and clinical work. As diverse functions of platelets in immune regulation have been recognized, they may also exert crucial roles in the development of fibrosis or inflammatory arthritis, which need to be elucidated in more future studies.

### Key Messages

Clinical characteristics of immune thrombocytopenia (ITP) associated with primary Sjögren's syndrome (pSS) have not been clearly defined.

This study revealed that secondary ITP was common in pSS patients with prevalence of 12.03% and have some distinct characteristics compared with pSS patients without ITP.

The prevalence of ILD and arthritis among pSS patients with ITP were significantly lower than pSS patients without ITP, indicating potential active roles of platelets in the pathogenesis of fibrosis or inflammatory arthritis.

## Data Availability Statement

The raw data supporting the conclusions of this article will be made available by the authors, without undue reservation, to any qualified researcher.

## Ethics Statement

The studies involving human participants were reviewed and approved by Clinical Research Ethics Committee of the First Affiliated Hospital of Xiamen University. The patients/participants provided their written informed consent to participate in this study.

## Author Contributions

YL and GS performed the study design. FD and GY analyzed data and wrote the manuscript. PR, PW, RC, YS, YP, HQ, BW, and SC collected and analyzed data. All authors approved the final manuscript.

## Conflict of Interest

The authors declare that the research was conducted in the absence of any commercial or financial relationships that could be construed as a potential conflict of interest.
